# Working hand syndrome: A new definition of non-classified polyneuropathy condition

**DOI:** 10.1097/MD.0000000000007235

**Published:** 2017-06-23

**Authors:** Gökhan Özdemir

**Affiliations:** Atatürk University Medical Faculty, Department of Neurology, Erzurum City, Turkey.

**Keywords:** carpal tunnel syndrome, hand-arm vibration, pain, working hand syndrome

## Abstract

The aim of this paper was to define an unexplained non-classified polyneuropathy condition as a new neurological disease. This new diagnosis of occupation related polyneuropathy has been named as “WORKING HAND SYNDROME (WHS).”

This study collected and compared clinic and electrophysiological analyze data from healthy controls, WHS patients, carpal tunnel syndrome (CTS) patients and polyneuropathy patients. The WHS patients presented to the clinic with pain, numbness, tingling, and burning sensations in their hands that increased significantly during rest and nighttime. However, there was no weakness in the muscles, and the deep tendon reflexes were normal in this disease. The patients had all been working in physically demanding jobs requiring the use of their hands/arms for at least 1 year, but no vibrating tools were used by the patients. All of the cases were men. I supposed that overload caused by an action repeated chronically by the hand/arm may impair the sensory nerves in mentioned hand/arm. In patients with these complaints, for a definitive diagnosis, similar diseases must be excluded. Nonetheless, the specific electrophysiological finding that the sural nerves are normal on the lower sides, as well as the occurrence of sensory axonal polyneuropathy in the sensory nerves without a significant effect on velocity and latency in the work-ups of the upper extremity are enough to make a diagnosis.

In conclusion, WHS has been defined as a polyneuropathy and occupational disease. Patients with WHS present with pain, numbness, tingling, and burning sensations in their hands that increases significantly during rest and nighttime. They also use their arms/hands for jobs that require heavy labor. The neurological examinations of patients with WHS are normal. Only the sensory nerves in the upper extremities are affected. This article is suggested to serve as a resource for patients, health care professionals, and members of the neurology community at large.

## 1 Introduction

Peripheral polyneuropathy has a prevalence of 2% in the general population. However, if there are one or more risk factors involved, this rate can increase to 12% to 17%.^[1]^ Various systemic diseases, exposure to toxicity, drugs, infections, and hereditary diseases are considered causes. Symptoms usually include numbness and paresthesia in the peripheral polyneuropathies. These symptoms are often accompanied by weakness and can be painful. Usually, there is a progression from distal to proximal. Diminished deep tendon reflexes, distal muscle weakness, and atrophy are common in advanced cases. Anamnesis, neurological check-up, and electrophysiological work-up are recommended for diagnosis.^[2,3]^ One of the most common causes of neuropathic pain in the hands is physical compression of the nerves, known as compression neuropathy. Carpal tunnel syndrome (CTS) and cubital tunnel syndrome are examples. Direct injury to a nerve, interruption of its blood supply, or inflammation may also cause neuropathic pain.

Electrophysiological work-ups show axonal damage (axonal neuropathy), demyelination (demyelinating neuropathy), and both (mix neuropathy). In the electrophysiological work-ups that involve distal latency, the amplitude, shape, and velocity of the motor and sensory nerves are checked. Axonal degeneration causes a decrease in amplitude, while demyelinating polyneuropathy causes delays in distal latencies and decreases in velocity. Acute axonal damage in the motor nerves can cause spontaneous activities in muscle fibers when checked with electromyography, where dilution in voluntary activity and chronic neurogenic motor unit potentials (MUP) are seen.

Two patients reported neuropathic pain in their hands, and axonal neuropathy was detected only in the sensorial neurons of the upper extremity. The common trait for both of these patients was the fact that they used their hands/arms during heavy labor. A literature review was conducted afterward, but no disease that fit the definition was identified. After similar patients were detected, these patients were recorded in their personality health file. I thought that a significant number of patients as this should not to be underestimated in the general population. Common traits among the patients include man sex, use of the arms and hands in heavy labor, neuropathic pain in their hands, and axonal polyneuropathy in the sensory median and ulnar nerves. My goal is to use the literature to discuss these cases, define a new disease, and exclude similar conditions.

## 2 Materials and methods

### 2.1 Subjects

The patients were recorded between 2015 and 2017 after applying to our policlinic and clinic (Ataturk University Medical Faculty, Department of Neurology, Erzurum City, Turkey). A hospital-based study was carried out on 10 subjects in total (Table 1). The average age of the patients was 45.7 ± 20.4 years. None of the WHS cases had systemic disease, and all of the cases were men.

**Table 1:**
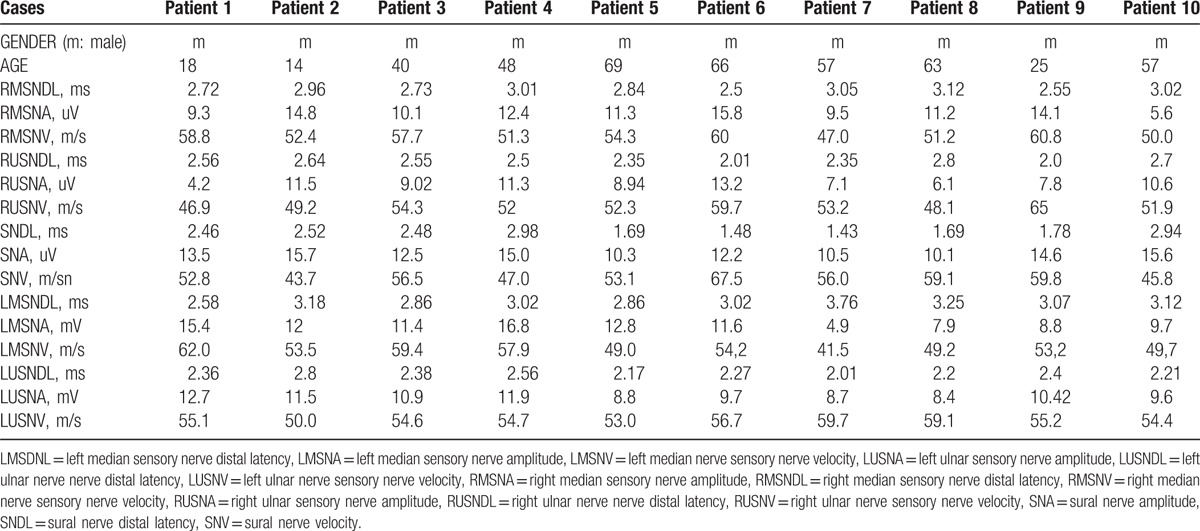
Age and electrophysiological findings of our patients.

### 2.2 Inclusion criteria

The use of the upper extremity while working a physically demanding job (construction worker, farmer, forester, crushing, tire repairer) requiring the use of the hands/arms for at least 1 year; presentation with pain, numbness, tingling and burning sensations (neuropathic) in the hands and fingers that increases significantly during rest and nighttime; and no presence of any diseases described in the literature.

### 2.3 Exclusion criteria

The use of a vibrating tool by the patients and the presence of a nervous system disease, such as polyneuropathy, CTS, or hand-arm vibration syndrome (HAVS).

### 2.4 Control groups

The control groups were created to make comparisons with my patients. The control groups were comprised of patients with polyneuropathy (n  =  10) and CTS (n  =  10), which may be confused with WHS. In these control groups, the motor nerves were unaffected. Similar to the WHS cases, the polyneuropathy and CTS cases were selected only if the sensory nerves have been affected and were men of a similar age group. In addition, 10 healthy individuals of the man gender, with no complaints, and of the same age group were included in the study. All of the groups were evaluated according to the electrophysiological, clinical, and examination findings.

### 2.5 Nerve conduction studies

The electrophysiological work-ups in my patients and in the control group patients were completed with standardized supramaximal percutaneous stimulation techniques using an 8-channel Neuro-EMG-micro (Neurosoft) device. In the upper sides, a sensorial check-up was completed of the median and ulnar nerves. The sural nerves were used for a lower extremity sensory evaluation. For the motor evaluation and the sensorial evaluation, surface and ring electrodes were used, respectively. For the median motor nerve evaluation, a 6 to 7 cm proximal of the abductor policies brevis muscle was supramaximally stimulated; the ulnar motor nerve was recorded from the abductor minimi muscle; the median sensorial nerve was recorded from the second finger, and the ulnar sensorial nerve was recorded from the fifth finger. For the sural nerves, the active electrode was placed between the lateral malleolus and the heel, and the reference electrode was placed 30 mm distally at the lateral edge of the foot. Supramaximal stimuli were applied at 13 cm proximal to the active electrode, just lateral to the midline of the calf. Amplitudes below 16 uV for the sensorial nerves in the upper sides and amplitudes below 10 uV for the sensorial nerves in the lower sensory sides (sural nerves) were considered the limits of sensory axonal neuropathy to assess its sensitivity and specificity. The use of an infrared lamp ensured that the temperature of the extremities during measurement would be 34 °C or higher.

Local ethics committee approved.

### 2.6 Statistical analyses

All numerical data, such as electrophysiological results or numerically scored parameters, were analyzed using the Duncan multiple comparison test, which allows for an inter-comparison of all groups (*P* < .05). For comorbid disease analyses, Fisher exact test was applied after the patient groups were combined. All results are expressed as mean ± SD for the patients in each group. The IBM statistics 20.00 program for Windows was used.

## 3 Results

### 3.1 Electrophysiological results

The electrophysiological findings of the WHS group and the control groups are presented in Tables 2–4. For the WHS group, according to the normal group, the distal latency and velocity of the median and ulnar sensorial nerves were similar in both hands. However, both the median sensory and ulnar sensory nerve amplitudes were decreased (*P* < .05). The motor nerve conduction work-ups of the upper and lower sides were similar in all 4 groups. The sural nerve results were similar on the lower sides in 3 groups (normal, CTS, and WHS). The sural nerve results were significantly affected in the polyneuropathy group (*P* < .05).

**Table 2:**
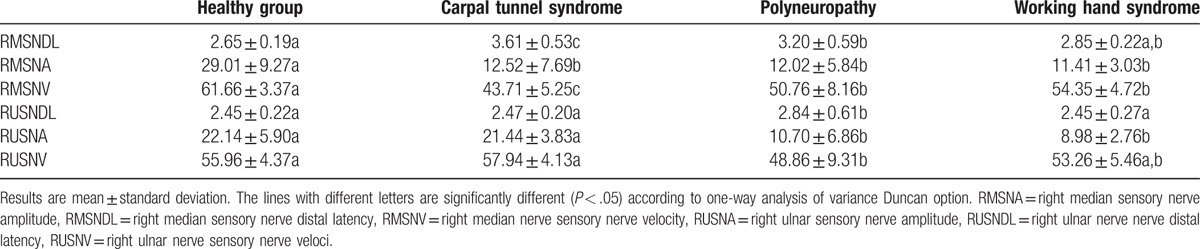
Right hand electrophysiological results.

**Table 3:**
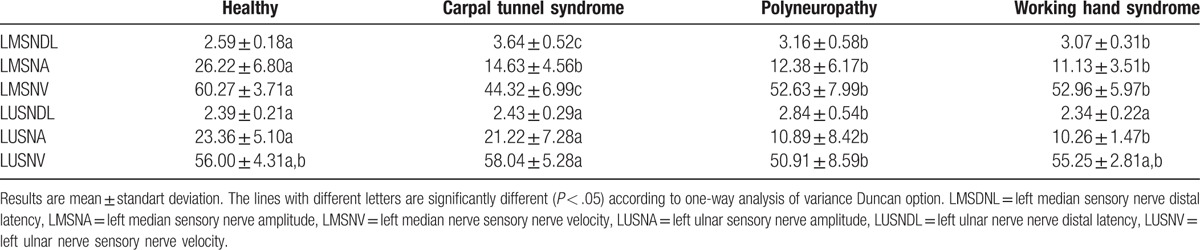
Left hand electrophysiological results.

**Table 4:**
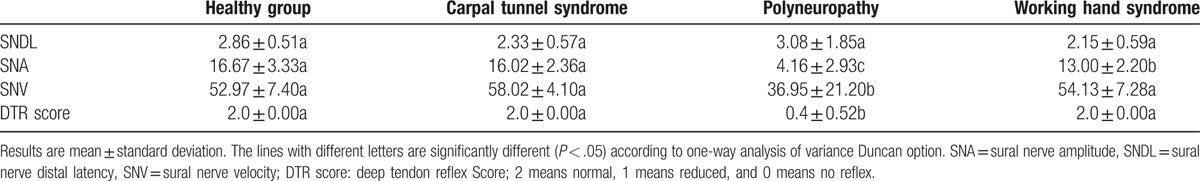
Sural nerve electrophysiological results and DTR score.

### 3.2 Clinical results

The deep tendon reflex polyneuropathy patients had a significantly decreased reflex when compared with all other groups (*P* < .05, Duncan). Regarding the presence of atrophy when all cases were compared with the WHS group, there was no significant difference according to Fisher exact test. In terms of hand complaints, the polyneuropathy group had a higher complaint score (1.3 ± 1.33; *P* < .05) when compared with the healthy normal group. However, the WHS (3.00 ± 0.00) and CTS (3.00 ± 0.00) groups exhibited an increase in hand complaints when compared with both the healthy and polyneuropathy groups.

### 3.3 Other comorbid diseases results

In terms of diabetes mellitus, hypertension, cardiovascular diseases, hyperlipidemia, cigarette smoking, and the presence of atrophy, when all cases were compared with the WHS group, there was no significant difference according to Fisher exact test. When the polyneuropathy group was compared with all other cases, there were no significant differences in terms of cardiovascular diseases, hyperlipidemia, cigarette smoking, and the presence of severe atrophy. For diabetes mellitus (*P*: .07) and hypertension (*P*: .015), the comorbidity in the polyneuropathy group differed significantly from that in the other groups, according to Fisher exact test.

## 4 Discussion

Pathology in the sensory nerves can cause neuropathic pain. Sensory polyneuropathy is one of the most common causes of neuropathic pain. The diagnosis of distal axonal sensory polyneuropathy is extracted from nerve conduction work-up reports based on the presence of bilateral, symmetric, distal lower, and upper extremity neuropathic pain. The motor nerves are unaffected, and there is no muscle weakness in this condition.^[2]^ Only the hands experienced neuropathic pain in the WHS group, while there was neuropathic pain in both the feet and hands in the polyneuropathy group.

Sensory nerve conduction work-ups of the median, ulnar, and sural nerves are widely used in the electrodiagnosis of sensory polyneuropathy. The long nerves are most commonly affected by polyneuropathy. Thus, the sural sensory nerve action potential (SNAP) amplitude is likely the most useful parameter for differentiating normal subjects from those with distal sensory polyneuropathy. Even the sural SNAP is most sensitive in the diagnosis of early distal sensory polyneuropathy.^[4]^ The sural nerve results were significantly affected in the polyneuropathy group, while the WHS group had normal sural nerve conduction work-ups.

Several diseases affect the nerves of the hand, the most common being CTS, which is caused by median nerves in the carpal tunnels becoming stuck. It is characterized by neuropathic complaints in the first 4 fingers and the palm of the hand. Its symptoms manifest usually during rest hours or nighttime, and the cases identified in this article were similar in that regard. This means the entirety of their hand and the fingers had neuropathic pain. Women are more commonly affected by CTS, and rheumatism, pregnancy, and diabetes are among the known risk factors for CTS. All of my cases were men, and they had no known CTS risk factors. Characteristic electrophysiological findings of CTS include a progressively delayed sensory peak latency, and amplitude becomes smaller in the median nerve. In medium cases, similar findings appear in the motor nerves. In advanced cases, SNAP and compound muscle action potential (CMAP) values decrease, which means that in CTS, a delayed distal latency and decrease in velocity are pronounced in the median nerve. The ulnar nerve conduction work-ups in CTS are normal.^[5–7]^ In this study, the ulnar sensory nerve conduction work-ups were normal, while the median sensory nerves were affected in the CTS group. In the WHS group, according to the normal group, distal latency and velocity were close to normal, but both the median sensory and ulnar sensory nerve amplitudes were decreased. Motor values were completely normal.

Guyon canal and cubital tunnel entrapment neuropathies can cause neuropathic pain, as well,^[8]^ but neuropathic pain is seen only in the ulnar nerve tract. In nerve conduction studies, distal latency and velocity are affected in the ulnar nerve. In all of the cases here in, neuropathic pain was identified in every region of the hand. Not only the ulnar nerve but also the median nerve was affected.

The mechanical energy created by vibrating tools, which enters the body through the fingers or palms, is called hand-arm vibration. These tools are generally used in the production, stone working, mining, construction, agriculture, and forestry sectors. HAVS is a clinical condition that occurs after exposure to hand-arm vibration. Symptoms of HAVS include numbness, pain, and reduced dexterity, strength, and sensation in the hands. In HAVS, the peripheral and central nervous systems are affected,^[5]^ which can lead to vascular, bone and joint, and tendon and muscle diseases.^[9,10]^ There is a direct correlation between the disease and the magnitude and duration of hand-arm vibration and cold temperatures.^[11]^ In the cases here in, no vibrating tools were used by the patients, but they did engage in taxing labor using their hands (using such tools as a sledgehammer, hammer, saw, and carry stones). It was argued that the usage of beta-blockers and cigarettes and a decrease in blood circulation due to exposure to the cold lead to an increase in HAVS symptoms.^[12,13]^ According to the anamnesis of the patients in this study, their symptoms did not change in cold temperatures or after smoking. CTS is often observed in people with HAVS who engage in breaking stones, plating, and forestry. This means that HAVS itself can cause CTS. Electrophysiological studies aimed at defining the nature of a vibration injury have provided conflicting results.^[14]^ Usually, electrophysiological findings related to HAVS are similar to those related to CTS, and the effect on velocity is pronounced. These conditions can be seen together often.^[15]^

The ulnar nerve is rarely affected in HAVS, but both the ulnar and median nerves were affected in our patients. Especially, the ulnar nerve was affected. In HAVS, slowed sensory nerve conduction velocities are often observed in the hands. In my cases, especially, the amplitude was low without being greatly affected by speed and latency. In vibration-associated neuropathies, conceivable target structures could be peripheral sensory receptors; large or thin myelinated nerve fibers; and small-caliber, non-myelinated C fibers. Pathological studies by cutaneous biopsy have demonstrated demyelinating neuropathy in the digital nerves of individuals with HAVS.^[16]^

It is believed that WHS is likely a sensory neuropathy with such a mechanism as axonal polyneuropathy, because the ulnar nerve is more affected than the median nerve in the upper extremities in polyneuropathies. I posit that an overload caused by an action repeated chronically by the hand/arm may impair the sensory nerves in said hand/arm. Not only the peripheral nervous system but also the local vessels may also be affected. This process may result in vasoconstriction of the local vessels. This situation leads to hypoxia and a lack of nutrition in the sensory nerves. However, there is not a clear relation between WHS and its pathology. However, in my opinion, genetics, ergonomics, emotional stress, and biodynamic status play an important role in WHS, because this disease does not occur in everyone who is doing the same job.

## 5 Conclusion

WHS is a polyneuropathy and occupational disease. Patients with WHS present with pain, numbness, tingling, and burning sensations in their hands that increases significantly during rest and nighttime. They also use their arms/hands for jobs that require heavy labor. The neurological examinations of patients with WHS are normal. Only the sensory nerves in the upper extremities are affected.

For a definitive diagnosis:

1. All have been working in physically demanding jobs requiring the use of the hands/arms.
2. Patients exhibit neuropathic pain in their hands.
3. The exclusion of similar diseases (Tables 5 and 6).
4. Specific electrophysiological findings that the sural nerves are normal, as well as the occurrence of sensory axonal polyneuropathy in the sensory nerves without being greatly affected by speed and latency in the work-ups of the upper extremity are enough to make a diagnosis.

**Table 5:**
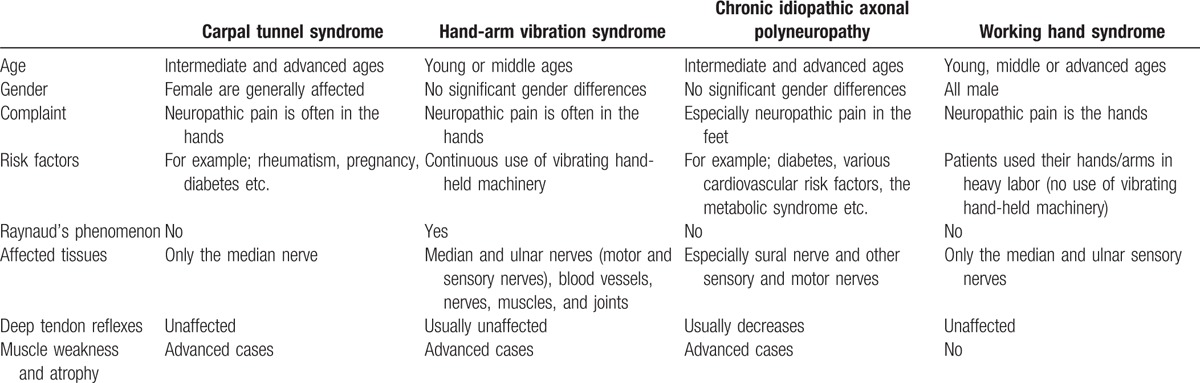
Differential diagnosis of working hand syndrome.

**Table 6:**
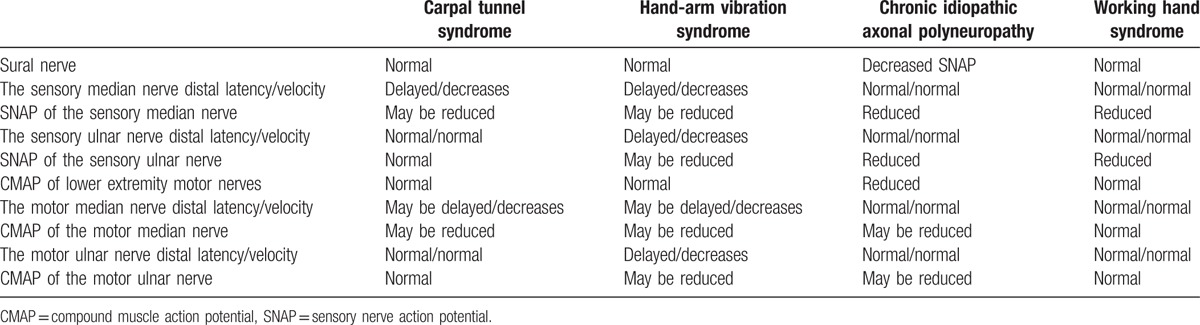
Electrophysiological findings of working hand syndrome and similar diseases.
